# A Case of Klippel-Trenaunay Syndrome With Gastrointestinal Hemorrhage and Splenomegaly

**DOI:** 10.7759/cureus.74371

**Published:** 2024-11-24

**Authors:** Tamzid Ahmed Pranta

**Affiliations:** 1 Medicine and Surgery, Chittagong Medical College, Chittagong, BGD

**Keywords:** congenital vascular malformation, gastrointestinal hemorrhage, klipper-trenaunay syndrome, limb hypertrophy, splenomegaly

## Abstract

Gastrointestinal bleeding resulting from the involvement of the gastrointestinal tract in people with Klippel-Trenaunay syndrome (KTS) is exceedingly uncommon and frequently neglected. A 22-year-old male, a diagnosed case of KTS, was assessed for per rectal bleeding and abdominal discomfort. A colonoscopy revealed third-degree hemorrhoids with vascular malformation all over the colon. Cystic lesions were found in the spleen with associated splenomegaly on ultrasonography. The patient was managed conservatively. Due to extensive colonic involvement, surgical procedures were not done with limited facilities. He was advised to take more specialized and expert consultations.

## Introduction

 Klippel-Trenaunay syndrome (KTS) is a rare congenital condition characterized by abnormal blood vessels primarily affecting the lower extremities. This condition is distinguished by the presence of capillary, lymphatic, and/or venous abnormalities and excessive growth of soft tissue and bone [1]. The prevalence of KTS is estimated to be one in 100,000 live births [2]. Involvement of the gastrointestinal tract is rare in patients with KTS, and about 1%-12.5% of cases exhibit involvement of the distal colon and rectum [3]. Splenic involvement in patients with KTS is rare [4] and could be due to hemangioma, lymphangioma, or both. In this report, we provide a case of a patient experiencing uncommon gastrointestinal hemorrhage, along with splenomegaly and cystic malformations in the spleen.

## Case presentation

**Figure 1:**
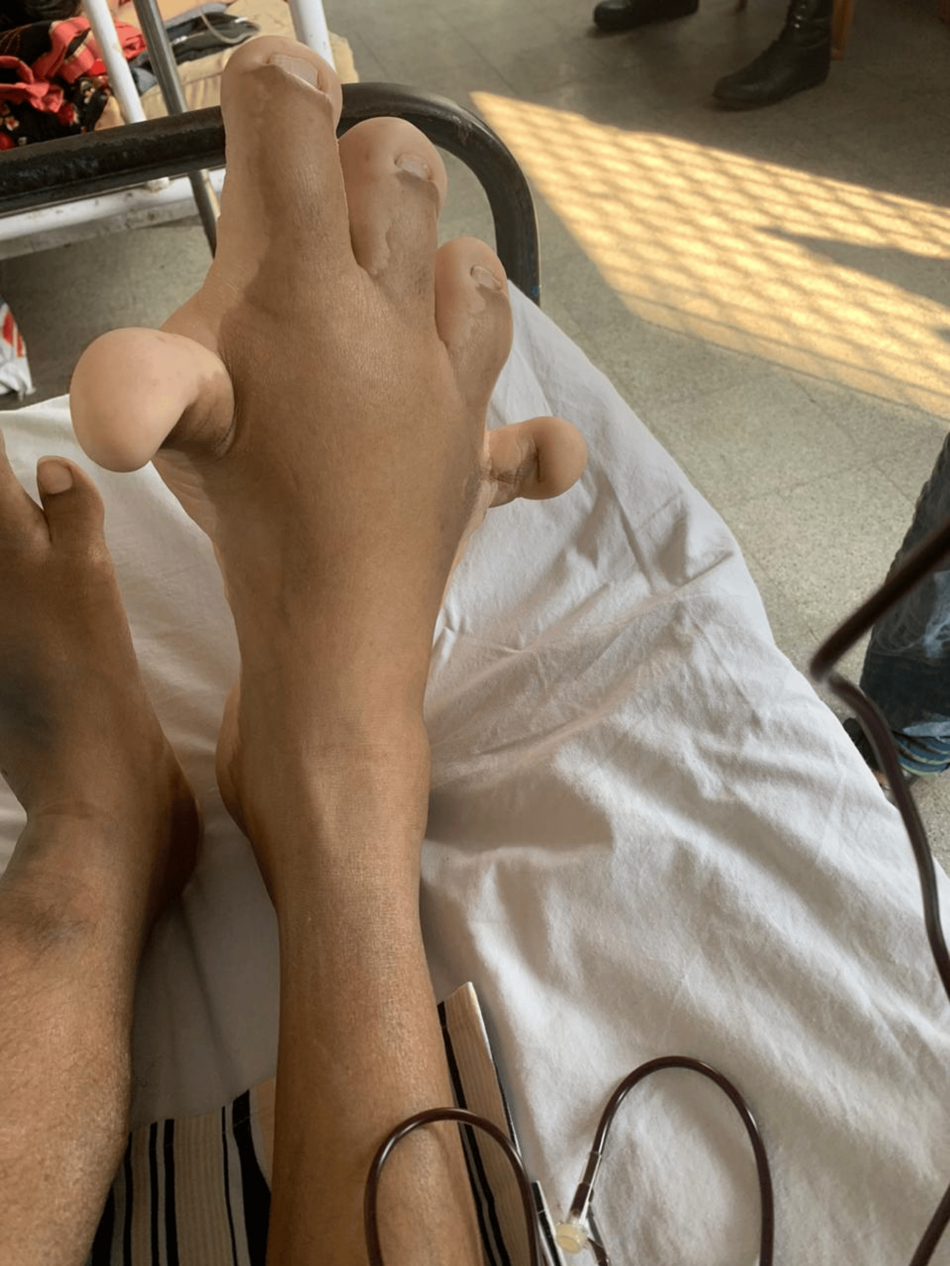
Hypertrophied right lower limb with extensive involvement of the foot.

A 22-year-old male with KTS was evaluated for abdominal discomfort and per rectal bleeding with associated anemia. He had been diagnosed with the syndrome in childhood. The history of the disease includes repeated hospitalizations for acute anemia requiring multiple transfusions since the age of 13 years. The patient had no history of allergies or family history of KTS. Physical examination revealed a hypertrophied right lower limb (Figure 1), which was extensive in the foot, with associated dilated veins.

On the color Doppler of the right lower limb, there were dilated, torturous venous channels and saphenofemoral incompetency in the right lower limb. But there was no arterio-venous fistula found.

**Figure 2:**
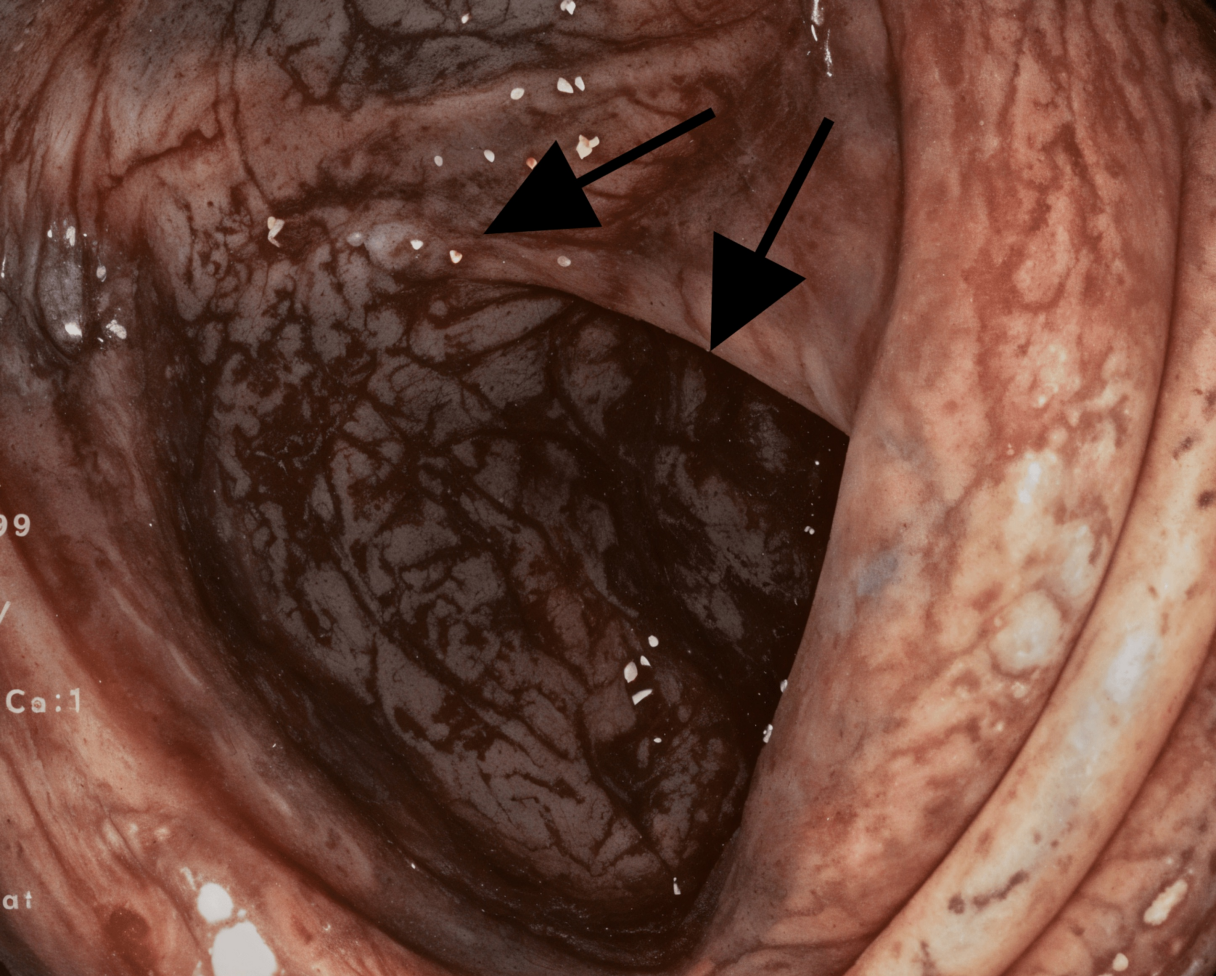
Inflamed, erythematous vascular malformations involving all over the colon (black arrows).

On colonoscopy, third-degree hemorrhoids were found. A colonoscopy was done up to the caecum and the whole mucosa was inflamed, edematous, erythematous, ulcerated, and friable with vascular malformations all over the colon (Figure 2). Due to the appearance of the varicosities, biopsies were not taken for fear of uncontrollable bleeding from the vascular malformation. 

**Figure 3:**
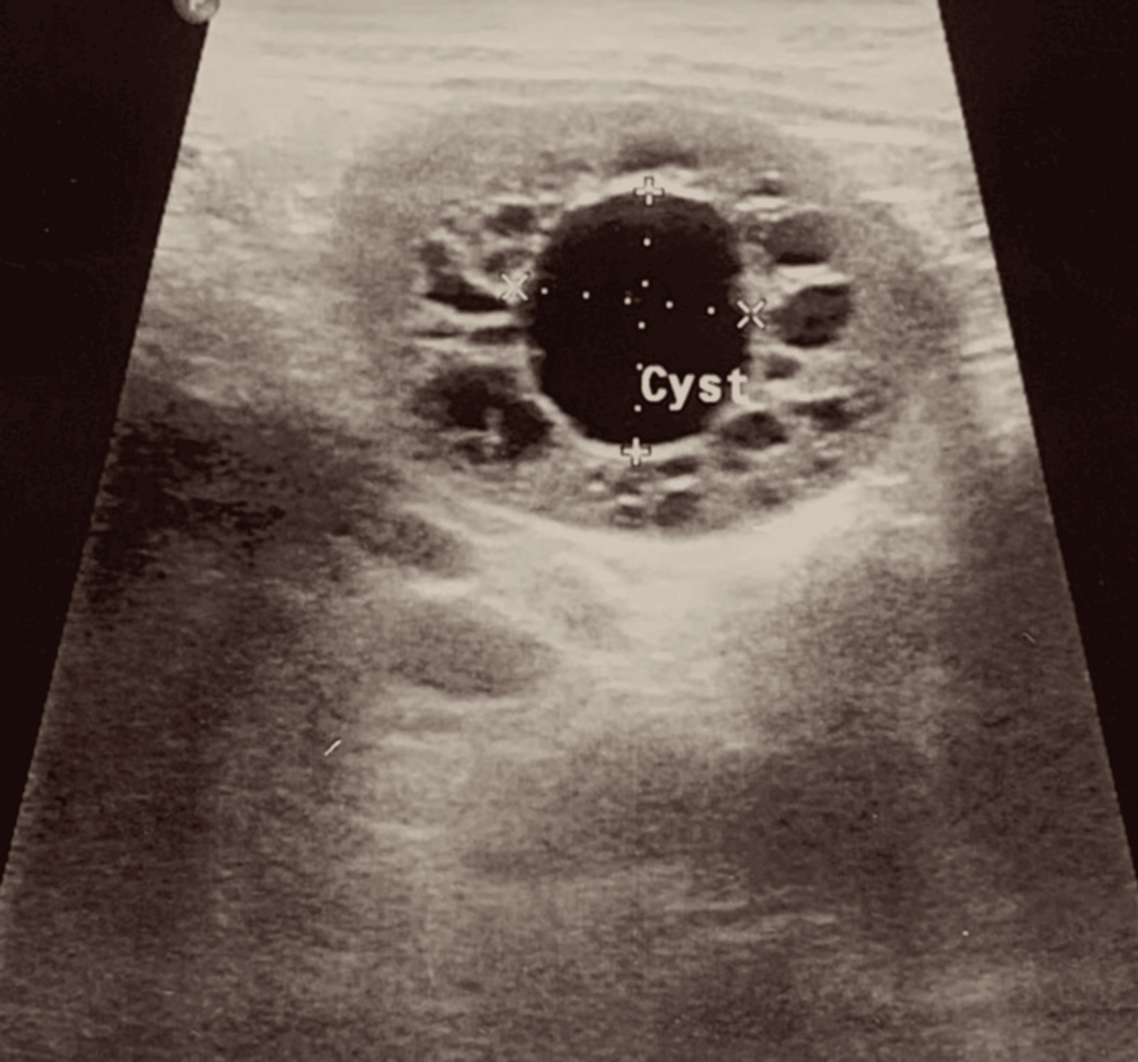
Cystic malformations in the spleen.

On ultrasound of the whole abdomen, all other organs were normal except the enlarged spleen. It was about 13.6 cm in size and multiple cystic structures were noted in the spleen, the largest one measuring about (1.8*1.7) cm (Figure 3).

Due to his extensive colorectal involvement of vascular malformations, no surgical procedures were done due to a lack of specialized approach and expertise. He was treated conservatively with elastic compression stockings and oral folic acid and transfused with three units of packed cell volume on admission. He was advised to keep regular follow-ups for blood transfusion purposes and seek more expert consultation regarding further surgical management.

## Discussion

Signs of KTS often manifest either at birth or during childhood, and most cases are sporadic [5]. One possible explanation for KTS is that abnormal angiogenesis is caused by a fatal gene (*PIK3CA*) that survives through somatic mutations or mosaicism [6]. The sigmoid colon and rectum are the most affected areas in the gastrointestinal tract. The clinical signs of this condition can vary from being asymptomatic to causing severe gastrointestinal bleeding that can be life-threatening [7,8]. Anorectal vascular malformation bears a resemblance to hemorrhoids in terms of its appearance; however, it is not a genuine case of hemorrhoids. The presence of malformation and reflux is typically indicative of this condition [9]. The presence of “hemorrhoids” may indicate the existence of gastrointestinal involvement and/or portal hypertension in KTS, which is merely the beginning of a larger problem. Hence, it is advisable to refrain from using unselective sclerotherapy as a means to treat “hemorrhoids” [9]. Treatment of gastrointestinal bleeding has proven to be challenging. Endoscopic therapies have a limited role due to the extensive nature of the disease, as in our patients. A conservative approach with iron replacement may be used for nonsignificant bleeding. Endoscopic treatments, such as endoscopic laser therapy and endoscopic clipping [3], can be used to treat specific, confined areas of damage or injury [10]. Patients with greater extent and severe symptoms of this condition may undergo surgical resection, which consists of a sphincter-preserving excision of the sigmoid colon and rectum that are involved, followed by a colon pouch-anal anastomosis and protective loop ileostomy [8,11]. Splenic lesions in KTS patients are characterized by the presence of multifocal or diffuse lymphatic abnormalities and Splenectomy may be recommended for cases of severe splenomegaly [12]. The research gap of our study was not to distinguish histologically the splenic lesion. His gastrointestinal vascular malformation was so extensive that it was not possible to do surgical procedures with our limited facilities and expertise. He was advised then to seek more expert consultation for further management, whether if it is possible in Bangladesh or abroad.

## Conclusions

KTS is associated with vascular malformations, and it can involve the gastrointestinal tract that may present as per rectal bleeding. According to the involved area as well as well-equipped and expert consultations are needed before making decisions on surgical procedures, as there are risks of massive hemorrhage which can be life-threatening.
